# Pre-clinical evaluation of CYP 2D6 dependent drug–drug interactions between primaquine and SSRI/SNRI antidepressants

**DOI:** 10.1186/s12936-016-1329-z

**Published:** 2016-05-17

**Authors:** Xiannu Jin, Brittney Potter, Thu-lan Luong, Jennifer Nelson, Chau Vuong, Corttney Potter, Lisa Xie, Jing Zhang, Ping Zhang, Jason Sousa, Qigui Li, Brandon S. Pybus, Mara Kreishman-Deitrick, Mark Hickman, Philip L. Smith, Robert Paris, Gregory Reichard, Sean R. Marcsisin

**Affiliations:** Military Malaria Research Program, Experimental Therapeutics Branch, Walter Reed Army Institute of Research, 503 Robert Grant Ave, Silver Spring, MD 20910 USA

**Keywords:** Primaquine, Antimalarial, Drug metabolism, Pharmacokinetics, CYP 2D6, Relapsing malaria, Drug–drug interactions

## Abstract

**Background:**

The liver-stage anti-malarial activity of primaquine and other 8-aminoquinoline molecules has been linked to bio-activation through CYP 2D6 metabolism. Factors such as CYP 2D6 poor metabolizer status and/or co-administration of drugs that inhibit/interact with CYP 2D6 could alter the pharmacological properties of primaquine.

**Methods:**

In the present study, the inhibitory potential of the selective serotonin reuptake inhibitor (SSRI) and serotonin norepinephrine reuptake inhibitor (SNRI) classes of antidepressants for CYP 2D6-mediated primaquine metabolism was assessed using in vitro drug metabolism and in vivo pharmacological assays.

**Results:**

The SSRI/SNRI classes of drug displayed a range of inhibitory activities on CYP 2D6-mediated metabolism of primaquine in vitro (IC_50_ 1–94 μM). Fluoxetine and paroxetine were the most potent inhibitors (IC_50_ ~1 µM) of CYP 2D6-mediated primaquine metabolism, while desvenlafaxine was the least potent (IC_50_ ~94 µM). The most potent CYP 2D6 inhibitor, fluoxetine, was chosen to investigate the potential pharmacological consequences of co-administration with primaquine in vivo. The pharmacokinetics of a CYP 2D6-dependent primaquine metabolite were altered upon co-administration with fluoxetine. Additionally, in a mouse malaria model, co-administration of fluoxetine with primaquine reduced primaquine anti-malarial efficacy.

**Conclusions:**

These results are the first from controlled pre-clinical experiments that indicate that primaquine pharmacological properties can be modulated upon co-incubation/administration with drugs that are known to interact with CYP 2D6. These results highlight the potential for CYP 2D6-mediated drug–drug interactions with primaquine and indicate that the SSRI/SNRI antidepressants could be used as probe molecules to address the primaquine-CYP 2D6 DDI link in clinical studies. Additionally, CYP 2D6-mediated drug–drug interactions can be considered when examining the possible causes of human primaquine therapy failures.

**Electronic supplementary material:**

The online version of this article (doi:10.1186/s12936-016-1329-z) contains supplementary material, which is available to authorized users.

## Background

Malaria is one of the most common parasitic infections that negatively impacts global health and economic stability. Despite years of effort to control malaria, and recent efforts towards malaria eradication, malaria remains a global health threat [1–3]. Primaquine, an 8-aminoquinoline (8AQ) derivative, is the only drug licensed for the treatment of relapsing species of malaria (*Plasmodium vivax* and *Plasmodium ovale*) and has gametocytocidal activity which may reduce transmission of all parasites that cause human malaria. Despite the importance of primaquine in malaria treatment efforts, the mechanism of primaquine efficacy and haemolytic toxicity are not completely understood [4]. Recently, a series of studies indicated that primaquine is a pro-drug that is converted to an active metabolite(s) via hepatic metabolism [5–13]. CYP 2D6 and MAO-A are the key enzymes associated with primaquine metabolism, however, primaquine’s anti-malarial activity is dependent on CYP 2D6-mediated activation [6, 7, 12, 14, 15]. These recent series of discoveries are problematic for primaquine usage as human CYP 2D6 is highly polymorphic and metabolizes a significant portion of clinically used drugs [16, 17]. The involvement of CYP 2D6 metabolism with other drug classes could be problematic for primaquine because of the potential for CYP 2D6-mediated drug–drug interactions [18, 19].

Selective serotonin reuptake inhibitors (SSRIs) and selective serotonin and norepinephrine reuptake inhibitors (SNRIs) have been used effectively for the treatment of major depressive disorders, anxiety disorders, hot flashes, and post-traumatic stress disorder (PTSD) [20]. They have also been extensively characterized in regard to CYP 2D6 inhibition and drug–drug interactions. These classes of antidepressants are relevant to the general public, military service members, and travellers for the treatment of the various conditions listed above [21]. The interactions between primaquine and SSRI/SNRIs in this context have never been investigated with regard to the effect on primaquine metabolism. This is of interest because the SSRI and SNRI classes of antidepressant drugs have been extensively characterized for their potential to interact/inhibit CYP 2D6 both in vivo and in vitro (see Table 1 for a list of SSRI/SNRI CYP interactions) [22]. The thorough characterization of CYP 2D6-SSRI/SNRI interactions makes these molecules a good model chemical series for primaquine DDI interaction studies [22]. In previous reports, SSRI/SNRI inhibition of human CYP 2D6 has led to significant clinical alterations in pharmacokinetics/pharmacodynamics of co-prescribed drugs that are CYP 2D6 substrates [23, 24]. In that regard, the SSRI/SNRI antidepressants were used as tools in examining the impact of CYP 2D6-mediated DDIs in the context of primaquine pharmacology. Such analysis has been done with other drugs that are metabolized by CYP 2D6. An example is the anti-cancer drug, tamoxifen. Metabolism of tamoxifen by CYP 2D6 to its active metabolite (endoxifen) was altered by co-medication with paroxetine [25, 26]. Since efficacy of primaquine has been linked with CYP 2D6 metabolism, drugs that inhibit CYP 2D6 (such as the SSRI/SNRIs) could result in reduced primaquine anti-malarial efficacy and pharmacokinetic alterations.

**Table 1:** List of antidepressants utilized in this study

Antidepressant	Enzymes involved in biotransformation	Enzymes inhibited by antidepressant
Citalopram	CYP2C19, CYP2D6, and CYP3A4	CYP2D6 (weak)
Escitalopram	CYP2C19, CYP2D6, and CYP3A4	CYP2D6 (weak)
Fluoxetine	CYP2D6, CYP2C9, CYP2C19, and CYP3A4	CYP2D6 (strong), CYP2C9 (moderate), CYP2C19, (weak to moderate), CYP3A4 (weak to moderate), CYP1A2 (weak)
Fluvoxamine	CYP1A2 and CYP2D6	CYP2D6 (weak), CYP1A2 (strong), CYP2C19 (strong), CYP2C9 (moderate), CYP3A4 (moderate)
Paroxetine	CYP2D6 and CYP3A4	CYP2D6 (strong), CYP1A2 (weak), CYP2C9 (weak), CYP2C19 (weak), CYP3A4 (weak)
Sertraline	CYP2C9, CYP2C19, CYP2D6, and CYP3A4	CYP2D6 (weak to moderate), CYP1A2 (weak), CYP2C9 (weak), CYP2C19 (weak), CYP3A4 (weak)
Venlafaxine	CYP2D6 and CYP3A4	CYP2D6 (weak)
Desvenlafaxine	CYP3A4	CYP3A4 (weak)

Shown are the enzymes responsible for metabolism of each antidepressant, and enzymes each that each molecule inhibits. Table was adapted from [22]

## Methods

### Materials

Mixed-gender, pooled, cryopreserved, primary, human hepatocytes and cell culture reagents were purchased from BioreclamationIVT (Baltimore, MD, USA). The SSRI antidepressants (paroxetine, fluoxetine, fluvoxamine, sertraline, citalopram, escitalopram), and SNRI antidepressants (venlafaxine, desvenlafaxine) were purchased from Sigma Aldrich (St Louis, MO, USA). Recombinant human CYP450 2D6 and the NADPH regeneration system solutions A and B were obtained from BD Biosciences (San Jose, CA, USA).

### IC_50_ determinations

Recombinant human CYP 2D6 metabolism of primaquine was determined in the presence or absence of varying concentration of each antidepressant (0–100 μM) listed in the Table 1. Primaquine metabolism studies with human CYP 2D6 were conducted according to the manufacturer’s instructions. CYP 2D6 was pre-incubated with each corresponding antidepressant for 15 min prior to addition of primaquine. After primaquine addition (final concentration of 1 μM), aliquots were collected at 0 and 60 min for primaquine incubations, followed by quenching with an equal volume of acetonitrile. Sixty-minute incubations were chosen for IC_50_ determinations because of the robust primaquine metabolism signal observed on this timescale as previously reported (>80 % [27]). The samples were centrifuged at 15,700×*g* at 4 °C for 10 min, supernatants collected and analysed by liquid chromatography/tandem mass spectrometry (LC–MS/MS). IC_50_ values were determined from plots of per cent primaquine remaining against antidepressant concentrations. GraphPad (La Jolla, CA, USA) Prism 6 software was used for data analysis and graph plotting.

### Metabolism studies with CYP 2D6 and inhibition via fluoxetine

Metabolism studies with recombinant human CYP 2D6 were conducted according to the manufacturer’s instructions. Briefly, 10 μl of fluoxetine stock solution (stock-1 mM in DMSO) was mixed with the NADPH regeneration system buffers A (50 μl) and B (10 μl). Thirty microlitre of CYP 2D6 isoenzyme and 0.1 M phosphate buffer (pH 7.4) was added to bring the volume in each well to 960 μl. The solution was gently mixed by pipetting and incubated at 37 °C for 15 min. Ten microlitre of primaquine was then added to each well and the plate incubated at 37 °C for the duration of the experiment. The final primaquine and fluoxetine concentrations were 1 and 10 μM, respectively. Aliquots were collected at 0, 15, 30 and 60 min, followed by quenching with an equal volume of acetonitrile. The samples were vortexed for 30 s, and centrifuged at 15,700×*g* at 4 °C for 10 min. Supernatant was collected and immediately analysed by LC–MS/MS.

### Primary human hepatocyte culture

Cryopreserved, pooled, human, primary hepatocytes were flash thawed in a 37 ^°^C bath for 1 min. One vial of the thawed hepatocytes (5 million cells/vial) was added to 40 ml of pre-warmed thawing buffer, mixed thoroughly by gentle pipetting, and centrifuged at 72×*g* for 6 min. The supernatant was then aspirated and the pellet resuspended in hepatocyte incubation buffer (InVitroGRO HI media, BioreclamationIVT, Baltimore, MD, USA). The hepatocytes were then seeded onto 12-well culture plates, approximately 7 × 10^5^ hepatocytes/ml per well, and incubated in a humidified 5 % CO_2_/95 % air incubator for 30 min prior to the addition of compounds.

### Metabolism studies with pooled hepatocytes

Primaquine metabolism with primary, human, hepatocyte culture was conducted according to the methods published by Jin et al. [5]. Antidepressant compounds (final concentration of 10 μM) were pre-incubated with hepatocyte culture plates for 15 min prior to treatment with primaquine at 37 °C. Following the addition of primaquine (final concentration of 1 μM), aliquots (150 μl) were collected at 0 and 240 min; 240-min incubations were chosen for hepatocyte studies because of the robust primaquine metabolism signal observed on this timescale as previously reported (~40 % [5]). All reactions were stopped by adding an equal volume of acetonitrile. Samples were thoroughly mixed by vortexing and centrifuged at 15,700×*g* at 4 °C for 10 min. The supernatant was collected and 200 μl of sample was loaded on to a 96-well LC–MS/MS plate for analysis. Samples were analysed immediately after sample collection.

### Analysis by LC–MS/MS

Analyte peaks were detected and quantified using a TSQ Quantum triple quadrupole mass spectrometer (Thermo Fisher Scientific, Waltham, MA, USA). Mass spectrometry conditions were optimized for each analyte (see Additional file 1 for masses and ion transitions). Chromatographic separations were achieved using a Waters XTerra MS C18 (2.1 × 50 mm, 3.5 μm) column (Milford, MA, USA) with a Phenomenex (4 × 20 mm, 3.5 µm) guard column (Torrance, CA, USA) at a flow rate of 0.35 ml/min. A 7-min linear gradient from 5 to 95 % acetonitrile (0.1 % formic acid) was utilized. Samples were analysed with electrospray ionization in positive ion mode (source temperature 350 °C) by selected reaction monitoring at a collision pressure of 1.0 mTorr. Analyte peaks were detected and quantified using the Xcalibur software package.

### Pharmacokinetic measurements

All primaquine pharmacokinetic measurements were conducted as previously described in [14, 28]. Briefly, male, 9–12-weeks old, wild-type, C57BL/6 mice (Taconic Biosciences, Germantown, NY, USA) were used for pharmacokinetic evaluations. On arrival, animals were acclimated for 7 days in quarantine. The animals were housed in a cage maintained at a temperature range of 18–26 °C, 34–68 % relative humidity and a 12-h light/dark cycle. Food and water were provided ad libitum during quarantine and throughout the study. The animals were fed a standard rodent maintenance diet.

Pharmacokinetic studies were performed using oral administration. For primaquine dosing alone, three male C57BL/6 mice were dosed with primaquine at 20 mg/kg. The 20-mg/kg dose was chosen for this study because it has been extensively characterized previously in mice [14, 29]. For the primaquine + fluoxetine, mice received three doses of fluoxetine (4 mg/kg/day) for 3 days. The mouse fluoxetine dose of 4 mg/kg was chosen as this corresponds to the human equivalent of 20 mg tablets per day based on body surface area (0.32 mg/kg in humans). This dose has been shown to be safe and effective for fluoxetine in clinical trials. On the third day, primaquine (20 mg/kg) was co-administered with the last fluoxetine dose. The multiple day dosing regimen was chosen to reflect the repeated daily dosing of antidepressants in humans. Both primaquine and fluoxetine were reconstituted in water, and administered at 5 μl/g. For each timepoint, animals were euthanized and plasma and liver samples collected. Plasma was obtained from whole blood (500 μl) collected by cardiac puncture. Five-hundred microlitre of 1000 USP units/mL heparin (Hospira, Lake Forest, IL, USA) was added to whole blood samples to prevent coagulation prior to plasma isolation via centrifugation. Isolated plasma samples were stored at −80 °C until LC–MS/MS analysis. Liver samples were extracted from mice for each timepoint and immediately preserved on dry ice and stored at −80 °C until homogenization, extraction and LC–MS/MS analysis. Liver samples were resuspended using a sixfold dilution of ddH_2_0 (w/v) and homogenized by sonication. Liver samples were then extracted (1:1 v/v) with acetonitrile. The samples were vortexed for 30 s, and centrifuged at 15,700×*g* at 4 ^°^C for 10 min. Supernatant was collected and analysed by LC–MS/MS analyses.

### Primaquine anti-malarial efficacy experiments

Primaquine anti-malarial efficacy experiments were essentially conducted as described in [27, 29]. Briefly, oral primaquine and fluoxetine suspension solutions were prepared in ddH_2_0. Primaquine was dosed (20 mg/kg/day) using a three consecutive day treatment regimen (−1, 0, +1 day relative to infection). For the fluoxetine co-administration experiment, fluoxetine was dosed (4 mg/kg/day) 2 days prior and on every day of primaquine administration (i.e., −3, −2, −1, 0, +1 day relative to infection). Drug suspensions were made and transferred to a 20-ml bottle, drawn into a 1-ml syringe, and delivered via oral feeder (18 gauge) to the designated recipient. The *Plasmodium berghei* sporozoites used for inoculation (luciferase expressing) were obtained from laboratory-reared female *Anopheles stephensi* mosquitoes from Department of Mosquito Biology, Walter Reed Army Institute of Research, as described in [29]. Both liver and blood stages of *P. berghei* infections were monitored using the in vivo imaging system (IVIS) and flow cytometry as described in [29].

## Results

### Inhibition of CYP 2D6 mediated metabolism of primaquine by SSRI/SNRI drugs in vitro

**Fig. 1:**
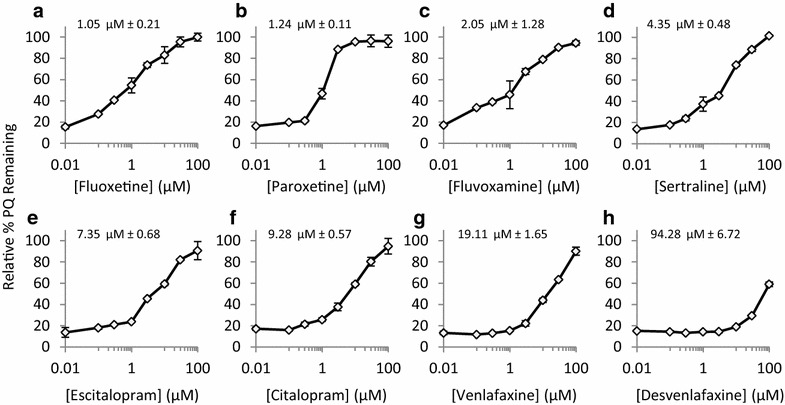
Inhibitory effects of second-generation antidepressants on primaquine metabolism by recombinant CYP2D6. The relative per cent primaquine remining after 60-min incubations with CYP 2D6 vs antidepressent concentration is shown for each compound tested. Fluoxetine (**a**) and paroxetine (**b**) were the most potent inhibitors for CYP2D6-mediated metabolism of primaquine, while desvenlafaxine (**h**) was the least potent. **c**–**g** are titration *curves* for the remaining antidepressants tested (fluvoxamine, sertraline, escitalopram, citalopram, venlafaxine). The determined IC_50_s are shown above each *graph*. The error shown is from duplicate experiments

Eight commercially available antidepressants were utilized to test the drug–drug interactions with primaquine. The antidepressants from the SSRI and SNRI classes chosen are shown in Table 1 (fluoxetine, paroxetine, fluvoxamine, sertraline, escitalopram, citalopram, venlafaxine, desvenlafaxine). The inhibitory potency of SSRI/SNRI compounds on CYP 2D6-mediated metabolism of primaquine were examined in vitro. Primaquine metabolism by recombinant CYP 2D6 isoenzyme was monitored after 60 min in the absence or presence of increasing concentrations of each SSRI/SNRI compound. SSRI/SNRI compounds were incubated with CYP 2D6 prior to primaquine addition. The results are shown in Fig. 1 as relative per cent of primaquine remaining after 60 min as a function of SSRI/SNRI compound concentration. The results illustrate that as the SSRI/SNRI concentrations increased, the relative per cent of primaquine remaining also increased suggesting CYP 2D6 inhibition and a reduction of primaquine metabolism. The IC_50_ values for each SSRI/SNRI incubation were determined by fitting each titration curve and are indicated for each SSRI/SNRI compound above each graph. The most potent inhibitors were fluoxetine and paroxetine with corresponding IC_50_ values of 1.05 µM ± 0.21 and 1.24 µM ± 0.11, respectively (Fig. 1a, b). The remaining SSRI molecules (fluvoxamine, sertraline, escitalopram, citalopram) had IC_50_ values of 2.05 µM ± 1.28, 4.35 µM ± 0.48, 7.35 µM ± 0.68 and 9.28 µM ± 0.57, respectively (Fig. 1c–f). Primaquine metabolism was least affected by the SNRI compounds venlafaxine and desvenlafaxine IC_50_ values of 19.11 µM ± 1.65 and 94.28 µM ± 6.72, respectively (Fig. 1g, h).

### Inhibition of CYP 2D6-mediated metabolism of primaquine by SSRI/SNRI drugs in hepatocytes

**Fig. 2:**
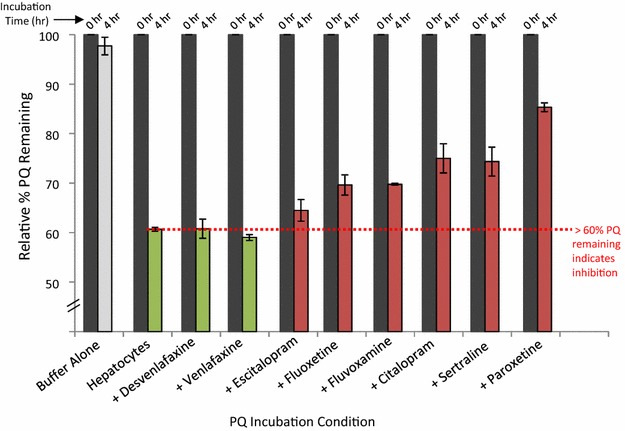
Primaquine metabolism by primary human hepatocyte culture pretreated with antidepressants (SSRI or SNRI). Samples were analysed by LC–MS/MS for primaquine disappearance. The 0 and 4-h incubation timepoint measurements for primaquine are shown for the controls and with each tested antidepressant. A relative per cent remaining value of <60 % for primaquine indicated no inhibition of metabolism (*green bars*) while a value of >60 % indicated inhibition by the indicated antidepressants (*red bars*). The error shown is from duplicate experiments

Having established the inhibitory trends of the different SSRI/SNRI molecules using recombinant CYP 2D6, SSRI/SNRI inhibition of primaquine metabolism was further assessed in primary human hepatocytes. Cell cultures were pre-treated with SSRI or SNRI drugs (10 µM) prior to primaquine incubations. Ten micrometre was chosen as a screening concentration to determine if each SSRI/SNRI had an effect on primaquine metabolism in hepatocytes. Primaquine metabolism results in hepatocytes are shown in Fig. 2. The relative per cent primaquine remaining is shown at 0 h (grey bars) and after 4-h incubation with hepatocytes. SSRI/SNRI drugs used are indicated on the x-axis. Primaquine was metabolized when incubated with hepatocytes with no SSRI/SNRI pre-treatment, as only 60 % of the primaquine remained after incubation period (green bar). This value was therefore used to determine if pre-treatment with the indicated SSRI/SNRI molecules caused inhibition of primaquine metabolism in the hepatocyte cultures when tested at 10 μM. The results for venlafaxine and desvenlafaxine indicated no inhibition of primaquine metabolism as primaquine levels after the 4-h incubation period were similar to the primaquine-hepatocyte only control. Pre-treatment of hepatocytes with the remaining antidepressant molecules resulted in decreased primaquine metabolism over the 4-h incubation period. These results are consistent with recombinant enzyme data and indicate that primaquine interacts in vitro with antidepressants that are CYP 2D6 inhibitors.

### Pharmacological interactions between primaquine and fluoxetine

**Fig. 3:**
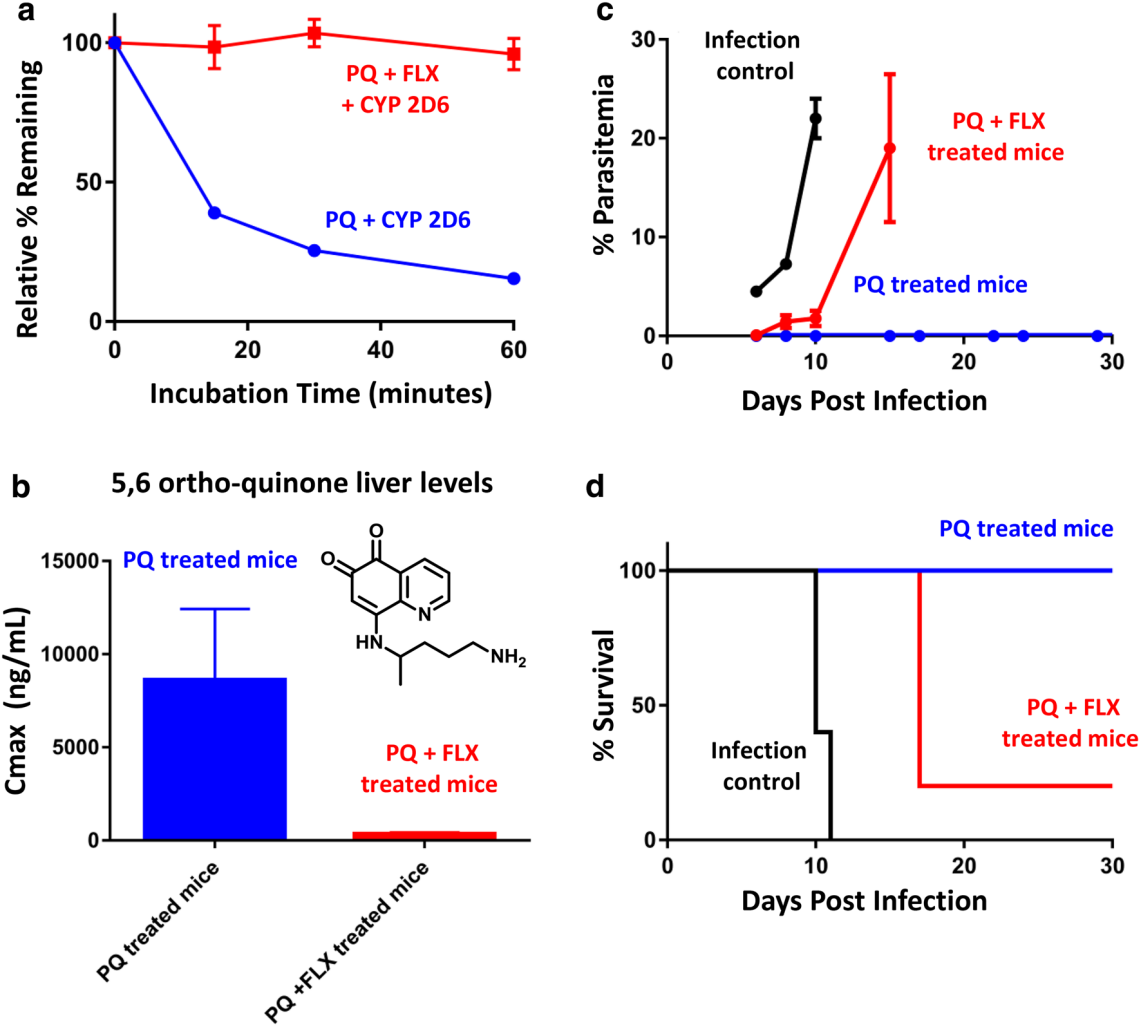
Pharmacological interactions between primaquine and fluoxetine. **a** Fluoxetine-mediated complete inhibition of primaquine metabolism by CYP 2D6 in vitro. Shown is the time course of primaquine CYP 2D6 metabolism alone (*blue line*), or upon fluoxetine pre-treatment (*red line*). The error shown is from duplicate experiments. **b** Liver Cmax values from pharmacokinetic analysis of the CYP 2D6-dependent primaquine metabolite 5,6-ortho-quinone from mice given a single primaquine dose (20 mg/kg, *blue bar*), or mice given a single primaquine dose (20 mg/kg) with fluoxetine (12 mg/kg daily for 3 days, see “Methods” section, *red bar*). The error shown is from triplicate pharmacokinetic analyses. **c** Average per cent parasitaemia measurements from flow cytometry measurement for the infection control group (*black line*), primaquine + fluoxetine (PQ + FLX)-treated mice (*red line*), and primaquine alone -treated mice (PQ, *blue line*). **d** Anti-malarial efficacy of primaquine in a prophylaxis mouse malaria model. The per cent survival of mice infected with *P. berghei* is shown for the different treatment groups. Mice infected and not given any anti-malarial drug are shown by the *black line* (0/5 survived duration of experiment). Mice infected and given primaquine at the ED_100_ are shown by the *blue line* (5/5 survived duration of experiment). Mice infected and given primaquine at the ED_100_ along with concurrent fluoxetine administration are shown by the *red line* (1/5 survived duration of experiment). Five mice were used per group for efficacy experiments

The results from the SSRI/SNRI IC_50_ determinations and hepatocyte experiments indicated that primaquine is likely prone to CYP 2D6-mediated drug–drug interactions when incubated with CYP 2D6 inhibitors. While insightful, the translation into a pharmacological effect for primaquine with CYP 2D6 inhibitor co-administration is currently unknown. The SSRI fluoxetine was chosen to investigate this as it was the most potent CYP 2D6 inhibitor tested (IC_50_ = 1.05 µM). The pre-incubation of CYP 2D6 with fluoxetine completely inhibited primaquine metabolism in vitro at 10 µM as shown in Fig. 3a. Fluoxetine is capable of reaching low micrometre concentrations in vivo and has a long elimination half-life [30]. Additionally, the major metabolite of fluoxetine (nor-fluoxetine) is also a potent CYP 2D6 inhibitor with a long elimination half-life. It was for these pharmacological reasons that fluoxetine was selected from the list of antidepressants to test and determine if co-administration with primaquine had any pharmacological effects.

The pharmacokinetics of primaquine and a CYP 2D6-dependent primaquine metabolite (5,6-orthoquinone) were determined in mice with and without fluoxetine co-administration. Interestingly, the pharmacokinetics of parent primaquine were not drastically altered by co-administration with fluoxetine (see additional files). The pharmacokinetics of the CYP 2D6-dependent metabolite (5,6-orthoquinone) were altered upon fluoxetine co-administration. The fluoxetine-treated mice had lower 5,6-orthoquinone liver concentrations as indicated in Fig. 3b, presumably from reduced CYP 2D metabolism as a result of a fluoxetine/CYP 2D-mediated drug–drug interaction. Reduced production of the 5,6-orthoquinone primaquine metabolite would imply that less of the active primaquine metabolite(s) would be produced in the fluoxetine-treated mice and thus might alter the anti-malarial activity of primaquine.

To ascertain if fluoxetine co-administration with primaquine had any effect on anti-malarial activity, a malaria mouse model employing *P. berghei* parasites was used to assess primaquine efficacy. This model has been used previously to characterize primaquine anti-malarial activity in the context of differential CYP 2D6 metabolism [27]. Primaquine was administered to mice at a therapeutic dose (20 mg/kg/day for 3 days) with and without co-administration of fluoxetine (4 mg/kg/day for 5 days, see “Methods” section). Presence of *P. berghei* infection was monitored via IVIS signal and flow cytometry. Out of the three groups tested (infection control, primaquine treated, and primaquine + fluoxetine treated), only the infection control had a robust IVIS signal during the first 72 h post infection. Blood stage parasitaemia measurements are shown in Fig. 3c and survival in Fig. 3d. Blood stage parasitaemia was detected in all mice of the infection control group. In the control group, 0/5 mice survived to the end of the experiment (day 31, see survival plot in Fig. 3d). Cure of *P. berghei* infection was defined as parasitaemia-free throughout the duration of the 31-day experiment. Three mice in the infection control group died on day 10 post inoculation and the remaining two were euthanized on day 15 due to high parasitaemia measurements. The primaquine-treated group (blue line) had 5/5 mice survive the duration of the experiment parasitaemia-free. The primaquine + fluoxetine-treated group (red line) had only 1/5 animals survive the duration of the experiment. The four mice that did not survive were euthanized on day 17 post inoculation due to high parasitaemia measurements (>5 %). The results from the primaquine + fluoxetine-treated mice indicate that even though a dose of primaquine was given that has been shown to be therapeutic against *P. berghi*, that co-administration of fluoxetine negatively impacted primaquine efficacy. These results along with the in vitro metabolism data presented above likely indicate that primaquine anti-malarial activity can be altered through CYP 2D6-mediated drug–drug interactions in mice.

## Discussion

The in vitro metabolism and in vivo mouse results above indicate the potential for drug–drug interactions between primaquine and the SSRI/SNRI antidepressants. While these results are insightful and suggestive, the clinical implications of such interactions remain unknown due to the limitations in extrapolating clinical outcomes from in vitro experiments and prophylactic mouse malaria models. In the study presented above, the in vivo effects of CYP 2D6-mediated DDIs with primaquine were only determined with fluoxetine at a fixed dose. Future studies using different CYP 2D6 inhibitors at different doses (to establish dose response correlations) are required to confirm that primaquine is susceptible to CYP 2D6-mediated DDIs. Controlled human clinical studies are ultimately needed to assess the impact of primaquine DDI interactions on anti-malarial efficacy as rodent malaria models are limited in that the antiparasitic activity of primaquine is only assessed for active hepatic schizonts and not dormant hypnozoites. The direct extrapolation of these results to human anti-malarial activity remains to be determined and warrants further investigation. In light of such limitations, it is interesting that the SSRI/SNRI inhibitory effects on CYP 2D6-mediated primaquine metabolism are similar to those measured using other CYP 2D6 probe substrates [31]. The IC_50_ values measured in vitro indicate the likelihood of primaquine-SSRI interactions with the most potent CYP 2D6 inhibitors (IC_50_ <2 μM, paroxetine and fluoxetine). SSRIs and other therapeutic agents (e.g., anti-malarial drugs that are also CYP 2D6 inhibitors) have been reported in the literature to have plasma concentrations in the mid-nanomolar to low-micromolar range [32] and would likely have elevated concentrations in the liver tissue where inhibition of hepatic metabolism would occur. It is also interesting to note that co-administration of fluoxetine with primaquine in the mouse malaria model did not completely inhibit the anti-malarial activity of primaquine. The primaquine–fluoxetine-treated group did not have detectable liver stage infection within the first 72 h of the experiment. Four out of five of these mice did have parasitaemia by day 8 post infection. Both the lack of IVIS signal within the first 72 h and delayed parasitaemia (in comparison to the infection control) for the primaquine–fluoxetine-treated mice indicates that fluoxetine reduces the anti-malarial activity of primaquine, however, it does not completely abrogate it. This phenomenon is probably a result of partial inhibition of the mouse CYP 2D pathway by fluoxetine. The effects of the primaquine–fluoxetine interaction in humans would likely be even more complex as the extent of fluoxetine-mediated CYP 2D6 inhibition would likely be different for the various human CYP 2D6 genotype/phenotypes.

Primaquine has been used for decades in the treatment of relapsing forms of malaria. Despite this, the mechanism of action for primaquine efficacy and clinical pharmacology in the context of drug–drug interactions is not fully understood. The recent advances in primaquine pharmacology regarding the requirement for CYP 2D6-mediated activation provide a possible explanation for some of the observed drug–drug interactions reported in the literature for primaquine [33, 34]. The work conducted by Purittayakamee et al., Hanboonkunupakarn et al. and Jittamala et al. highlight observed drug–drug interactions in humans between primaquine and the co-administered anti-malarials: chloroquine, dihydroartemisinin-piperaquine and pyronaridine-artesunate. All three combinations resulted in significantly higher primaquine exposure as a result of likely interactions with shared metabolic pathways. The human clinical significance of these interactions remains to be fully elucidated; it is interesting to note however these primaquine PK interactions in the context of the observed improved clinical outcomes reported for primaquine combination therapy back in 1955 by Alving and colleagues [35]. These findings along with the recent pharmacokinetic studies and the work by Alving et al. highlight the complex nature of primaquine drug–drug interactions and indicate that more than CYP 2D6 metabolism should be considered when interpreting such studies. Other factors, such as modulation of transporters and other CYP-metabolizing enzymes (other than CYP 2D6) should be taken into consideration for future primaquine studies. The primaquine-SSRI/SNRI work presented above is no different in that there could be other metabolic pathways modulated by co-administration of SSRI/SNRIs such as the CYP 3A4, 2C19 and/or CYP 2C9 as indicated in Table 1. Ultimately, more clinical observations are required to elucidate the human pharmacological consequences of primaquine drug–drug interactions in the context of anti-malarial activity.

## Conclusions

The worked presented above provides insight into potential drug–drug interactions with the anti-malarial drug primaquine. Additionally, this work brings to light another factor to consider when examining primaquine therapeutic failures. Successful primaquine therapy in humans seems to correlate with predicted CYP 2D6 phenotype. CYP 2D6 poor and intermediate metabolizers are more likely to fail therapy [7, 15, 36]. Potent CYP 2D6 inhibitors such as fluoxetine and other SSRI molecules have been shown using phenotypic analysis to significantly alter CYP 2D6 activity. Alfaro et al. showed that fluoxetine administration changed the CYP 2D6 phenotype of human subjects from extensive to poor metabolizers based on dextromethorphan/dextrorphan phenotypic analysis [24]. Such significant alterations of CYP 2D6 activity could have detrimental consequences for humans with vivax malaria undergoing primaquine therapy.

## Additional file

10.1186/s12936-016-1329-z Reference compounds utilized in Primaquine pharmacokinetic study. (A) The structures of Primaquine (PQ) and the marker for 5-hydroxylation (5,6-ortho-quinone) are shown. The liquid chromatography and mass spectrometry parameters utilized for quantitation of PQ and the phenolic metabolite. Shown are the observed m/z ions, product ms/ms ions, cone voltages, collision energies, and electrospray polarities utilized for quantitation. Additionally, the retention times for each molecule are also indicated. Separate standard curves and analyses of PK samples were conducted for each analyte. (B) Liver pharmacokinetics of a single 20 mg/kg dose of primaquine alone (blue line), or co-administered with fluoxetine (red line). (C) Liver pharmacokinetics of the 5,6-orthoquinone metabolite from a single 20 mg/kg dose of primaquine alone (blue line), or upon co-administration with fluoxetine (red line).

## Abbreviations

8AQ: 8-aminoquinoline
SSRI: selective serotonin reuptake inhibitor
SNRI: serotonin norepinephrine reuptake inhibitor
CYP: cytochrome P450
MAO: monoamine oxidase
PTSD: post-traumatic stress disorder
IC_50_: half maximal inhibitory concentration
DDI: drug–drug interaction
