# Supplementary material for: Pre-clinical evaluation of CYP 2D6 dependent drug–drug interactions between primaquine and SSRI/SNRI antidepressants
Source: Malar J. 2016 May 17;15:280. doi: 10.1186/s12936-016-1329-z (PMC4869338; doi:10.1186/s12936-016-1329-z)
Supplement: Supplementary file 1 — 10.1186/s12936-016-1329-z Reference compounds utilized in Primaquine pharmacokinetic study. (A) The structures of Primaquine (PQ) and the marker for 5-hydroxylation (5,6-ortho-quinone) are shown. The liquid chromatography and mass spectrometry parameters utilized for quantitation of PQ and the phenolic metabolite. Shown are the observed m/z ions, product ms/ms ions, cone voltages, collision energies, and electrospray polarities utilized for quantitation. Additionally, the retention times for each molecule are also indicated. Separate standard curves and analyses of PK samples were conducted for each analyte. (B) Liver pharmacokinetics of a single 20 mg/kg dose of primaquine alone (blue line), or co-administered with fluoxetine (red line). (C) Liver pharmacokinetics of the 5,6-orthoquinone metabolite from a single 20 mg/kg dose of primaquine alone (blue line), or upon co-administration with fluoxetine (red line). [file 12936_2016_1329_MOESM1_ESM.pdf]

A.

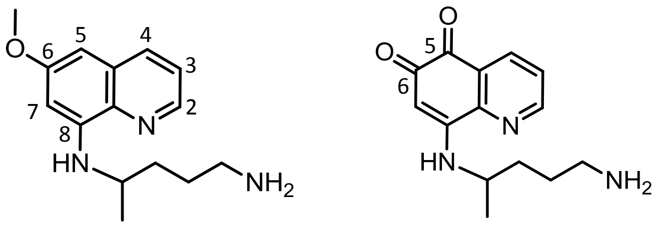


Primaquine

5,6-ortho-quinone

| | Observed Parent m/z | Product m/z | Cone (v) | Collision (v) | ESI Mode | Retention Time | Liver LLOQ (ng/mL) |
|---|---|---|---|---|---|---|---|
| PQ | 260.26 | 175.02 | 22 | 22 | Pos | 2.10 | 2.5 |
| 5,6-o-quinone-PQ | 260.20 | 147.08 | 26 | 30 | Pos | 1.80 | 5 |

B.

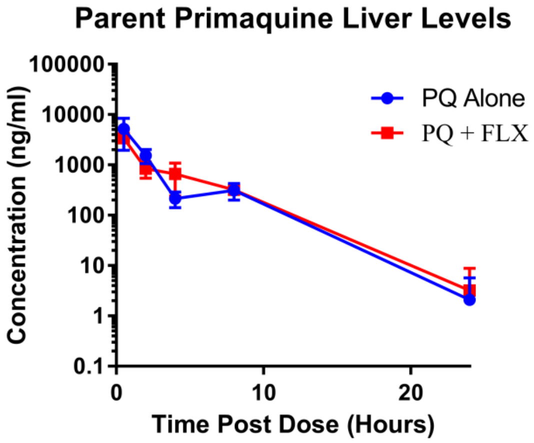


C.

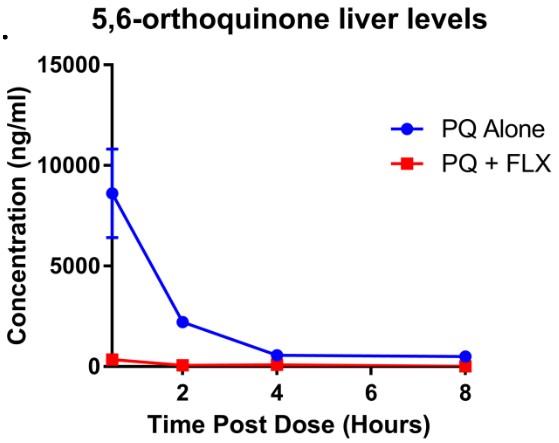


Additional Figure 1
